# Cells under pressure

**DOI:** 10.7554/eLife.68643

**Published:** 2021-04-23

**Authors:** Dhiraj Indana, Ovijit Chaudhuri

**Affiliations:** 1 Department of Mechanical Engineering, Stanford UniversityStanfordUnited States

**Keywords:** extracellular matrix, multicellular aggregates, mechanical stress, compression, tumor, cancer, Mouse

## Abstract

A new method for applying solid stress to aggregates of cells is shedding light on the impact of mechanical forces on cancer cells.

**Related research article** Dolega ME, Monnier S, Brunel B, Joanny JF, Recho P, Cappello G. 2021. Extra-cellular matrix in multicellular aggregates acts as a pressure sensor controlling cell proliferation and motility. *eLife* **10**:e63258. doi: 10.7554/eLife.63258

Packed like sardines, most cells in our body must operate in confined spaces. Neighboring cells, the extracellular fluid that surrounds cells, and the extracellular matrix that provides physical support to tissues, can all restrict the growth and motion of cells. This is exacerbated in tumors. As the cancer cells in the tumor multiply, they gradually push back the surrounding tissue, and in turn, experience varying degrees of compression or 'solid stress' (Nia et al., 2017). Increasing evidence suggests that the physical interactions between cancer cells and their microenvironment affect the signals that regulate their growth and spread.

Much of our knowledge about solid stress and its impact on tumor development stems from research on three-dimensional aggregates of cancer cells grown in the laboratory. When these aggregates – which are also known as multicellular tumor spheroids – multiply in a confined environment, such as a hydrogel, their growth is restricted (Helmlinger et al., 1997). These observations are somewhat intuitive, since cell division is an inherently physical process during which cells increase in volume and undergo striking morphological changes – both of which require space (Nam and Chaudhuri, 2018; Zlotek-Zlotkiewicz et al., 2015; Son et al., 2012).

It has been shown that restricting or reducing the volume of a cell through increased osmotic pressure – which draws water from the cell – stops them from multiplying (Nam et al., 2019; Delarue et al., 2014). Likewise, compression along one axis can suppress the growth of cells and even induce programmed cell death in tumor spheroids (Cheng et al., 2009). When applied transiently to single cells, it may also reverse the malignant phenotype, with the cells displaying behaviors of normal cells (Ricca et al., 2018).

However, full three-dimensional control of uniform compression of tumor spheroids has not been achieved to date. Now, in eLife, Giovanni Cappello, Pierre Recho and colleagues from the Université Grenoble Alpes, the Université de Lyon and the Collège de France – including Monika Dolega as first author – report a new way to study the impact of solid stress on tumor spheroids (Dolega et al., 2021).

Dolega et al. applied two strategies to compare the impact of solid stress on both single cells and tumor spheroids. To do so, they exerted osmotic pressure using osmolytes of varying sizes: small dextran molecules measuring less than 5 nm in diameter (following the standard approach), and large ones with a diameter of 15 nm or more (representing the new approach). The small dextran molecules were able to infiltrate the extracellular matrix of the spheroids, thereby applying osmotic pressure on the single cells within. However, the 15 nm dextran molecules were too big to enter the extracellular matrix: instead, the osmotic pressure acted on the entire spheroid, resulting in a global compression of the cells by the extracellular matrix (Figure 1).

**Figure 1.**
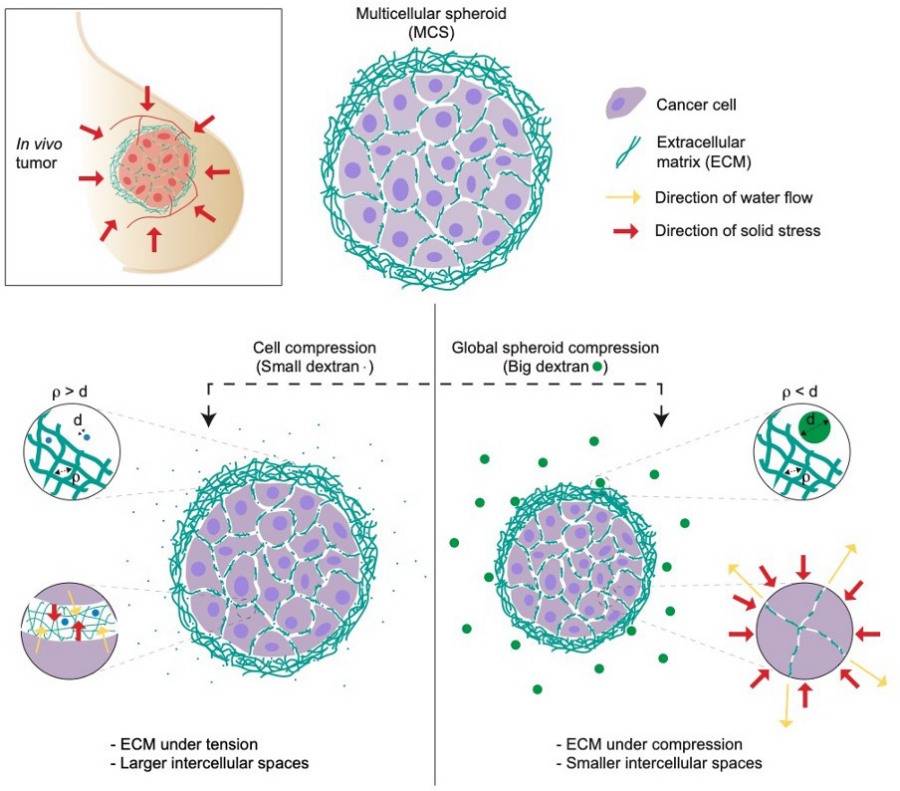
Studying the impact of solid stress on multicellular tumor spheroids. Multicellular spheroids (MCS) can be used as a model of in vivo tumors due to their ability to mimic several features of solid tumors (top left). To test the effect of solid stress on tumor growth, Dolega et al. used dextran molecules of varying sizes to induce osmotic water flow (yellow arrows). They found that small dextran molecules (blue circles, left) can enter the pores of extracellular matrix (ECM, green strings), thereby applying osmotic pressure (red arrows) on individual cells. Large dextran molecules (green circles, right) cannot enter the extracellular matrix, so they exert osmotic pressure on the whole spheroid (red arrows), mimicking the solid stress in in vivo tumors. d = diameter of dextran molecules; ρ is the spacing between the fibers in the extracellular matrix.

By keeping the magnitude of pressure the same, Dolega et al. discovered that global compression decreased the volume of the tumor spheroids significantly more than the osmotic compression of single cells, with cells within the spheroid growing and migrating more slowly. Comparable results were also observed in single cells encapsulated in an extracellular matrix-like hydrogel. There, global compression reduced the growth and migration of the cells while osmotic compression did not. These findings suggest that solid stress in the form of global compression does indeed regulate the growth and spread of a tumor.

Dolega et al. have developed a robust approach to probe the impact of solid stress on cells in three-dimensional microenvironments, and the different outcomes observed for osmotic compression of single cells and global compression of tumor spheroids draws attention to the possibility that the extracellular matrix can act as a pressure sensor that might regulate cell behavior. It remains unclear if cells sense solid stress through the same pathways that are implicated in osmotic compression, including stretch-activated ion channels, or through alternate pathways, perhaps involving the cytoskeleton and cell-matrix adhesions (Nam et al., 2019). Probing these mechanisms will help advance our understanding of how solid stress regulates cell behavior in a wide range of contexts, from embryonic development to cancer.
